# Targeting motor cortex high-excitability states defined by functional connectivity with real-time EEG–TMS

**DOI:** 10.1016/j.neuroimage.2023.120427

**Published:** 2023-12-15

**Authors:** David Emanuel Vetter, Christoph Zrenner, Paolo Belardinelli, Tuomas Petteri Mutanen, Gábor Kozák, Laura Marzetti, Ulf Ziemann

**Affiliations:** a Department of Neurology & Stroke, University of Tübingen, Tübingen, Baden-Württemberg, Germany; b Hertie-Institute for Clinical Brain Research, Tübingen, Baden-Württemberg, Germany; c CIMeC, Center for Mind/Brain Sciences, University of Trento, Trento, Trentino-Alto Adige, Italy; d Department of Neuroscience and Biomedical Engineering, Aalto Yliopisto, Espoo, Uusimaa, Finland; e Temerty Centre for Therapeutic Brain Intervention, Centre for Addiction and Mental Health, Toronto, Ontario, Canada; f Department of Psychiatry, University of Toronto, Toronto, Ontario, Canada; g Institute for Biomedical Engineering, University of Toronto, Toronto, Ontario, Canada; h Imaging and Clinical Sciences, Department of Neuroscience, University of Chieti-Pescara, Chieti, Abruzzo, Italy; i Institute for Advanced Biomedical Technologies, University of Chieti-Pescara, Chieti, Abruzzo, Italy

**Keywords:** Real-time, EEG, TMS, Corticospinal excitability, Motor network, Oscillations, μ-rhythm, Functional connectivity, Power, Phase

## Abstract

We tested previous *post-hoc* findings indicating a relationship between functional connectivity (FC) in the motor network and corticospinal excitability (CsE), in a real-time EEG-TMS experiment in healthy participants.

We hypothesized that high FC between left and right motor cortex predicts high CsE.

FC was quantified in real-time by single-trial phase-locking value (stPLV), and TMS single pulses were delivered based on the current FC. CsE was indexed by motor-evoked potential (MEP) amplitude in a hand muscle. Possible confounding factors (pre-stimulus μ-power and phase, interstimulus interval) were evaluated *post hoc*.

MEPs were significantly larger during high FC compared to low FC. Post hoc analysis revealed that the FC condition showed a significant interaction with μ-power in the stimulated hemisphere. Further, inter-stimulus interval (ISI) interacted with high vs. low FC conditions. In summary, FC was confirmed to be predictive of CsE, but should not be considered in isolation from μ-power and ISI. Moreover, FC was complementary to μ-phase in predicting CsE. Motor network FC is another marker of real-time accessible CsE beyond previously established markers, in particular phase and power of the μ rhythm, and may help define a more robust composite biomarker of high/low excitability states of human motor cortex.

## Highlights

• Functional connectivity (FC) was evaluated and targeted by real-time EEG-TMS.
• FC adds network perspective to EEG-TMS, complementing local measures (power, phase).
• Single-trial left M1–right M1 μ-oscillation FC predicts corticospinal excitability.

## 1 Introduction

The primary motor cortices (M1) do not operate in isolation, but are part of the bihemispheric motor network responsible for motor preparation and control. When moving, e.g., one hand, the contralateral M1 inhibits ipsilateral M1 (transcallosal, or interhemispheric, inhibition), and conversely, contralateral supplementary motor area and premotor cortex have excitatory projections to ipsilateral M1 (Rehme et al., 2011). The activity of the nodes in this network is modulated by the characteristic μ-rhythm. The μ-rhythm is an inhibitory brain rhythm in the 8–13Hz band, differing from occipital α (which covers the same frequency range) in being fully expressed when the eyes are open, but decreasing in power during motor activity or imagery (Garakh et al., 2020). It also differs in waveform from the more sinusoidal occipital α, as μ is markedly non-sinusoidal, with narrow troughs and broad peaks (arch-shaped wave, first described in Gastaut (1952)) (Garakh et al., 2020).

The role of this rhythm for motor control and excitability has been investigated locally, e.g. by relating the spectral power in the μ-band of a signal coming from M1 to motor excitability (Karabanov et al., 2021). Beyond the local perspective, it is important to study the activity of the whole motor network or of sub-networks (Grefkes and Fink, 2011). Measures of functional connectivity (FC) quantify this network activity, enabling us to investigate its relation to motor control and excitability. Measures of FC are intended to pick up the pairwise oscillatory interactions in a network (Bastos and Schoffelen, 2016). These oscillatory interactions form an functional network on top of and mediated by the structural connections in the network. They can thus serve to rapidly modulate the flow of information and implement communication between areas (as opposed to the slower-changing anatomical connectivity) (Fries, 2015). However, they do not indicate causal relations (Grefkes and Fink, 2011).

Transcranial magnetic stimulation (TMS) is a noninvasive brain stimulation method that suffers from high effect variability, both within and across subjects (Ziemann et al., 2019). This is especially true for open-loop stimulation (Ziemann et al., 2019), where stimuli are delivered irrespective of the current brain state. One line of current research thus seeks to identify opportune moments for stimulation determined by observable brain-states to achieve a desired effect (Zrenner et al., 2018, Baur et al., 2020). In brain-state-dependent stimulation, the TMS pulses are delivered only when such an opportune moment is observed in the ongoing electroencephalogram (EEG).

Corticospinal excitability (CsE) denotes the excitability of the cortico-spinal tract. When stimulating the hand representation area in M1 with TMS, CsE can be indexed by the amplitude of the motor evoked potential (MEP) as recorded in the electromyogram (EMG) of the contralateral hand.

The ongoing μ-rhythm modulates CsE: Both the spectral band power (Thies et al., 2018, Karabanov et al., 2021, Madsen et al., 2019) and the instantaneous phase of the μ-rhythm at the stimulated M1 have been found to be predictive of CsE (Hussain et al., 2019, Zrenner et al., 2018, Wischnewski et al., 2022). The effect of μ-phase on CsE does however depend on the μ-band power, in that during times of high μ-power, the trough of the μ-rhythm constitutes a high-excitability state, whereas for low power, the peak is the high-excitability state (Hussain et al., 2019). Power and instantaneous phase of the μ-rhythm therefore can be used to define opportune moments for stimulation, and have successfully been targeted in brain-state-dependent TMS experiments (Zrenner et al., 2018, Baur et al., 2020, Schaworonkow et al., 2018).

Additionally, a recent post-hoc analysis found measures of FC in the motor-network (left and right M1, supplementary motor area) in the μ-band to be predictive of CsE, where high functional connectivity indicated higher CsE (Marzetti et al., 2023). Beyond the μ-rhythm, the inter-stimulus interval preceding the stimulus is predictive of CsE — MEP amplitude increases with longer ISIs (Madsen et al., 2019), and the instantaneous phase of the β rhythm from the stimulated M1 also has been found predictive (Wischnewski et al., 2022).

In studying the motor network beyond a local perspective, the two-node network of left and right M1, which we also investigated here, was targeted in previous studies with bihemispheric TMS (Ferbert et al., 1992, Koganemaru et al., 2009, Rizzo et al., 2009, Stefanou et al., 2018). It has been demonstrated that the synchrony of the instantaneous phase of the μ-rhythm in the two M1s indicates the strength of interhemispheric communication between left and right M1 (Stefanou et al., 2018) – studying the μ-rhythm and its relation to TMS-effects is thus not only important with respect to predicting CsE excitability, but also to targeting cortico-cortical pathways.

Here, for the first time, we timed TMS based on a real-time estimate of μ-band FC, in a double-blind EEG–TMS experiment. We targeted left M1, to assess whether FC between left and right M1 could predict the CsE of the stimulated M1. We hypothesized that a high FC state would also be a state of high CsE.

In this paper, we will first give an overview over the experiment. Then, we will describe the details of the real-time signal-processing methods we used to time TMS on a measure of FC. We will then introduce the statistical analysis, both the *a priori* analysis, and the *post hoc* analysis. In the first part of the *post hoc* analysis, we will add pre-stimulus μ-power and inter-stimulus interval as confounders to the analysis, and formulate a summary model that gives an overview of these findings. In the second part of the *post hoc* analysis, we will assess the influence of volume conduction on our results, and relate our findings to the role of instantaneous μ-phase. The statistical analysis will be described in Methods and the statistical findings, including the summary model, will be given in the Results section.

## 2 Methods

We contrasted two conditions to highlight the influence of FC on CsE: The low condition was defined as a time when the current real-time μ-band FC estimate was below the lower quartile of an empirical distribution of the FC (see Sections 2.3, 2.5.3), and the high condition was a time when the current real-time μ-band FC estimate was above the upper quartile of the empirical distribution of the FC. The main hypothesis is, that stimulating in low condition yields smaller motor responses (derived from raw MEP) than stimulating in high condition.

### 2.1 Participants and inclusion criteria

The experiment conformed with the Declaration of Helsinki and followed current TMS safety guidelines (Rossi et al., 2021). The study protocol was approved by the ethics committee of the medical faculty at Tübingen university (project number 631/2021BO2). All participants gave written informed consent. They were paid 10€ per hour for participating in the experiments.

Originally, 16 healthy, right-handed participants were included in the study. However, one had to be excluded because they felt nauseous during stimulation (the session was immediately aborted and the participant was supervised until they no longer felt nauseous and felt ready to leave). Thus, 15 participants (age: 23.8 ± 2.4 years (range: 19–28), assigned sex: 6 female) were fully included. Before participation in this study, participants were screened for a sufficiently expressed μ-rhythm, required to get meaningful phase-estimates: Only participants with a peak of 5 dB above individually fitted $\frac{1}{f}$-noise, in the μ-range (8–13 Hz) of the signal coming from left somatosensory cortex (spatial filter: C3-Hjorth/surface Laplacian (Hjorth, 1975)) were included. About two thirds (65%) of screened participants had sufficient SNR.

Participants were not generally required to undergo an MRI, but if a subject-specific MRI was available, it was used for neuronavigation (individual MRI was used for 5 of 15 participants).

### 2.2 Materials

We used a 64-channel EEG-cap with TMS-compatible Ag/AgCl sintered ring electrodes (‘BrainCap TMS’, by EASYCAP GmbH, Woerthsee-Etterschlag, Germany). ‘Nuprep’ (Weaver and Company, Aurora, USA) abrasive gel and ‘Vyaire electrode cream’ (Vyaire Medical Oy, Helsinki, Finland) were used to prepare the skin-electrode interfaces. For the EMG, glue-on Kendall ECG Electrodes (CardinalHealth, Dublin, Ireland) were placed on the muscle belly of the right-hand *first dorsal interosseus* (FDI) and the proximal interphalangeal joint of the index finger, and the muscle belly of the *abductor pollicis brevis* (APB) and the thumb’s interphalangeal joint respectively (bipolar belly-tendon montage). EEG and EMG were jointly recorded at a sampling rate of 5 kHz using a Bittium NeurOne Tesla EEG System (Bittium Corporation, Oulu, Finland). Electrode impedances were generally brought under 5 kΩ: strictly so for C3/C4-Hjorth electrodes, but higher impedances were tolerated for peripheral electrodes, which are not of immediate interest to this study.

**Fig. 1:**
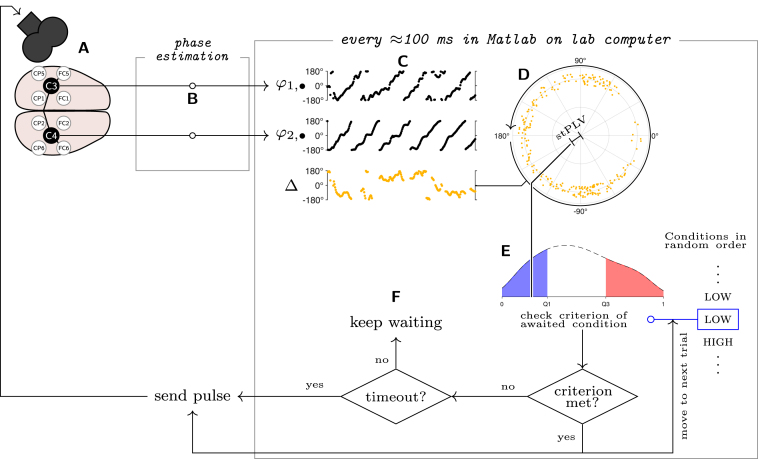
Overview of the closed-loop EEG-TMS setup: the signal from the C3- and C4-Hjorth montages was recorded (A), and processed in real time by the bossdevice (B), which yielded the estimated instantaneous phases from the last 500 ms (250 samples, $\varphi_{1,1},\ldots \varphi_{1,N},\varphi_{2,1},\ldots ,\varphi_{2,N}$ in Eq. (1)). The phase estimates were retrieved by the controlling PC, and the instantaneous phase differences (Δ) were computed (C). From these, the single-trial PLV was obtained as the magnitude of the complex-valued average of the phase-difference phasors (all of unit length, here jittered for readability; D). The current stPLV was then compared against the criterion of the current condition (E). In the visualized case, the current condition is low, so the current stPLV is compared to the lower quartile of the empirical stPLV-distribution. If the stPLV is below the low-condition threshold, a pulse was sent, followed by a 2 s pause (minISI=2 s), and the system proceeded to wait for the next condition (here: high). If the criterion was not met, the system checked whether the time since the last stimulus exceeded 8 s – if it did, a timeout pulse was sent, followed by the 2-s minISI pause — but the condition was not updated. If timeout had not been reached, the system simply kept waiting (F). When waiting, the 500 ms windows of phases were retrieved (roughly) every 100 ms. Every computed stPLV was also added to the empirical stPLV-distribution (E) after comparing it to the current criterion, replacing the oldest stPLV — then, the criteria were recomputed.

The real-time evaluation of the EEG-data was partially done on a commercial real-time EEG processing system (‘brain oscillation synchronized stimulation’ device/bossdevice prototype, sync2brain GmbH, 72076 Tübingen), on which an algorithm for real-time phase-estimation (Zrenner et al., 2020) was run (implemented in Simulink real-time; custom commercial software provided by sync2brain GmbH). The real-time phase-estimates were then streamed to a separate PC, on which the FC computations were performed (see Fig. 1) – as the commercial software on the real-time EEG processing system did not offer FC-support.

Biphasic TMS pulses were delivered using a ‘MagPro XP’ stimulator (MagVenture A/S, Farum, Denmark) and an actively cooled figure-of-eight coil (MagVenture Cool-B65 coil by MagVenture A/S, Farum, Denmark).

The subject’s head position and the coil’s position were tracked using a stereoscopic neuronavigation system (Localite GmbH, Sankt Augustin, Germany). The head position was supported and fixed using a vacuum neck support pillow (Vacuform, B. u. W. Schmidt GmbH, Germany).

### 2.3 Experimental procedure

Each participant was subjected to two identical sessions, each consisting of preparation (≥1 h), resting state EEG (8–9 min), and two stimulation blocks (≈25 min each) with a 10 min break in between. Sessions were at least two days, and at most 3 weeks apart.

After EEG and EMG preparation, the subject was instructed to stay awake and at rest, while seated in the experimenting chair. The subject was instructed to fixate the fixation target displayed on a screen in front of the chair (distance to eye: 1.5 m), blink as little as possible, and not close their eyes during measurements. When we observed muscle artifacts in the C3-/C4-Hjorth EEG-data before the resting state or stimulation blocks, the participant was specifically instructed to relax facial muscles, and shown their EEG from the C3- and C4-Hjorth montage (spatially filtered, and individual channels), to help them get the relevant muscles relaxed. During measurements, participants were instructed by messages displayed on the screen to relax their face and/or hand, if muscle artifacts were noticeable in the EEG/EMG — but they were not shown their EEG or EMG in these cases.

The motor hotspot was manually identified as the coil position and rotation giving the highest MEPs in the FDI muscle, and yielding MEPs at the lowest stimulation intensities (Rossini et al., 2015). This motor hotspot was registered in the Localite neuronavigation software. The resting motor threshold (RMT) was then manually identified as the minimum stimulator intensity that yielded a visually recognizable MEP for 50% of stimuli delivered at the hotspot. Additionally, we determined an MEP-input–output(IO)-curve, delivering 5 pulses at 100%, 110%, 120%, 130%, and 140% RMT respectively. This acted as a safeguard, to make sure that the stimulation intensity (110% RMT) was well within the dynamic range of the IO-curve, and not under- or over-estimated (avoid saturation).

During all EEG recordings (resting state and EEG–TMS blocks), subjects listened to a customized masking noise (generated with TAAC (Russo et al., 2022)) over in-ear headphones. Before the recordings, the volume (and ear-plug placement) was individually adapted so that the participant could no longer hear the TMS coil click next to (but off) their head, while keeping the volume safe and bearable.

In the resting state EEG recording, an initial empirical FC- distribution was acquired, comprising 1000 individual estimates of FC, computed approximately every 500 ms from a window of 500 ms of data (Basti et al., 2022). This initial distribution served to define the initial values of the condition criteria (upper and lower quartile).

In the stimulation part of each session, 450 low and 450 high condition trials were measured. The order of the conditions was randomly permuted (interleaved) at the beginning of each session and remained unknown to the subject and experimenter until after the experiment. The number of high and low condition trials was approximately balanced between the two stimulation blocks (450 trials each). Stimuli were delivered at 110% RMT, with a minimum inter-stimulus interval (ISI) of 2 s.

### 2.4 Spatial filters

To observe the effects of the sensorimotor μ-rhythm on CsE, a generic surface Laplacian filter centered on the C3-electrode (as defined by the international 10–20 system (Seeck et al., 2017)) has been found to yield an informative signal (when stimulating left M1) (Zrenner et al., 2018, Schaworonkow et al., 2018, Karabanov et al., 2021). This spatial filter assigns a weight of 1 to the center-electrode C3, and weights of $-\frac{1}{4}$ to the diagonally adjacent electrodes FC1, FC5, CP1 and CP5 and is called C3-Hjorth (after (Hjorth, 1975)). C4-Hjorth is the corresponding spatial filter for the right M1, centered on C4. We thus used two generic spatial filters for all subjects, C3-Hjorth for the left hemispheric signal, and C4-Hjorth for the right hemispheric signal.

### 2.5 Quantifying functional connectivity in real-time

#### 2.5.1 Single-trial functional connectivity

To quantify functional connectivity between two nodes (here: left and right M1), we used a single-trial version of the phase-locking value (stPLV) between the two signals: For a time-window of N=250 phase-estimates $\varphi_{1,1},\ldots ,\varphi_{1,N}$ and $\varphi_{2,1},\ldots ,\varphi_{2,N}$ from those two signals (here: C3-/C4-Hjorth signal), the stPLV is computed as the magnitude of the mean of the unit-length phasors of the phase-differences in the window (Lachaux et al., 1999):

(1) $$\mathrm{stPLV}=\left|\frac{1}{N}\overset{N}{\underset{k=1}{\sum }}e^{i\left(\varphi_{1,k}-\varphi_{2,k}\right)}\right|$$

StPLV is ≈1 if there is a consistent phase difference between the two signals in a given single trial, and near zero if there is no consistency in the phase difference. StPLV differs from regular PLV in that we here take an average over time, not over trials (Bastos and Schoffelen, 2016). Such a stPLV has been described and characterized by Basti et al. (2022). It has previously been estimated that a data window covering about 5 cycles of the rhythm of interest – i.e. a window of 500 ms for μ – is suitable to capture the dynamics of phase-coupling in this frequency range (Ermolova et al., 2021, Basti et al., 2022). We thus used N=250 real-time estimates of instantaneous phase, because the employed real-time EEG-processing system provided phase-estimates at 500 Hz sampling rate. PLV (and stPLV) is a very simple measure of functional connectivity based on phase consistency, which importantly is undirected, and can be inflated by volume conduction.

Additionally applying the imaginary-part operator I before the magnitude-operator yields the imaginary part of stPLV (istPLV) instead:

(2) $$\mathrm{istPLV}=\left|I\left(\frac{1}{N}\overset{N}{\underset{k=1}{\sum }}e^{i\left(\varphi_{1,k}-\varphi_{2,k}\right)}\right)\right|$$

IstPLV ignores any phase-locking of zero or 180° phase-shift, and ignores the supposed zero-phase-shift component of non-zero phase-locking. Such 0/180° phase-locking may be the result of a common source being picked up in two different electrodes (volume conduction artifact) (Nolte et al., 2004). The mean of the phasors before taking the absolute value or imaginary part will here be called the ‘complex stPLV’ (cstPLV).

#### 2.5.2 Estimating instantaneous phase in real time

The instantaneous phase of the ongoing μ rhythm was estimated using a commercial real-time EEG processing system, which ran the phastimate-algorithm of Zrenner et al., 2018, Zrenner et al., 2020 which is adapted from the work of Chen et al. (2011). The algorithm receives a window of ongoing EEG-activity as its main input (and algorithm parameters as secondary inputs), and estimates the instantaneous phase at the end of this window (the timepoint of interest). In the real-time setting, it receives the most recent second of spatially filtered EEG data, and estimates the instantaneous phase at present. To this end, the window of data is first filtered into the frequency band of interest (with zero-phase-shift filtering). Then, the edges are cut off from this window of filtered data, to get rid of edge-artifacts that would distort the phase estimation at the end of the window. On the remaining window, an autoregressive (AR) model is fit. This AR model is then used to forecast the oscillation, beyond the timepoint of interest. The analytic signal around the timepoint of interest is estimated using the Hilbert transform, and the instantaneous phase at the timepoint of interest is retrieved from the analytic signal.

#### 2.5.3 Implementation of real-time FC estimation

To define the high and low conditions, we needed real-time estimates of the stPLV, and to test our hypothesis, we then needed to send TMS pulses when an awaited condition occurred (‘trigger on the condition’). For this, we used the real-time EEG processing system and the accompanying commercial closed-source firmware, and complemented it with dedicated customized software implemented in Matlab 2017b, running on a separate PC. The real-time EEG processing device provided a 1-s window of real-time phase-estimates from up to two spatially filtered signals. Based on this, the presence of either condition was evaluated on the PC, and pulses were then triggered from the PC.

We configured the real-time EEG-processing device to estimate the instantaneous phase of the μ-rhythm (8–15 Hz; based on the observed peaks in the C3-Hjorth periodograms) of the C3- and C4-Hjorth-spatially-filtered signals, with a sampling-rate of 500 Hz. During the resting-state EEG, the PC retrieved the window of the most recent 500 ms of phase-estimates (most recent 250 samples) from the C3/C4-Hjorth signals roughly every 500 ms (non-overlapping windows) and computed the current stPLV, until it had collected 1000 stPLVs. This process took about 8–9 min. These 1000 stPLVs then served to define the initial values of the lower and upper quartile criteria for the stimulation.

For the stimulation blocks, the order of the conditions (450 low, 450 high) was randomly permuted. The system then went through the list of conditions one by one, triggering a TMS stimulus when the current condition was met. After sending out a stimulus, the system paused for 2 s (minimum ISI), and then waited for the next condition. If the time since the last pulse exceeded 8 s, a ‘timeout’ pulse was delivered, another 2 s were paused, and then the wait for the current condition was resumed. This was done to avoid dishabituation, and especially not give the participants the impression that the stimulation block was over. The process during the stimulation blocks is visualized in Fig. 1. Timeout pulses do not belong to either condition and were thus ignored during the analysis.

##### Details.

When waiting for a condition, the PC retrieved the 500ms-windows of phase-estimates of the C3- and C4-Hjorth-signals from the bossdevice roughly every 100 ms (overlapping windows, otherwise the same as in resting state), computed the stPLV, and compared it to the lower quartile if waiting for the low condition (upper quartile if waiting for the high condition). If the condition was met, a pulse was triggered (and the minimum ISI enforced). When a pulse (except time-out pulses) was triggered, the phase-estimates that yielded the stPLV were saved into a log-file. Whether a pulse was triggered or not, the new stPLV was also added to the list of 1000 samples from the stPLV-distribution, and the oldest stPLV was removed, keeping the list at 1000 entries. The quartiles of the empirical distribution were then updated (dynamic condition criteria, see supplementary Fig. S3). StPLVs were not estimated during the 2 s after each pulse, to avoid the influence of transcranially evoked potentials (TEPs) on the stPLV-distribution.

**Fig. S1:**
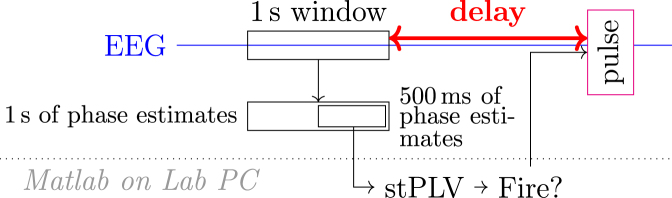
The delay of interest is between the right edge of the stPLV-window and the stimulus being delivered. The time of the pulse is directly marked in the NeurOne-recording.

**Fig. S2:**
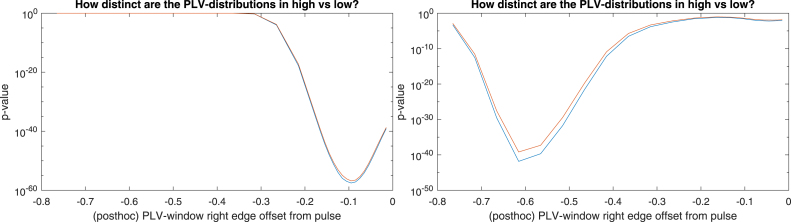
p-values of the one-sided Mann–Whitney-U-test checking whether the *post hoc* stPLVs in high condition are higher than those in the low condition, for different stPLV-window-offsets (orange: relative to upper criterion, blue: relative to lower): Left: The lowest p-value is achieved for 85 ms. Towards the pulse (0 lag), the separation of the conditions is still high (p$\approx 10^{-40}$), but much weaker than for this offset. Right: Example of the result for the incorrect first implementation: An artificial 500 ms delay was introduced by using the wrong end of the window of phase-estimates (first half in Fig. S1).

Because the PC was responsible for retrieving and evaluating the phase estimates, and for sending the triggers to the stimulator via the real-time EEG-processing system, there was a fluctuating delay in this setup: Stimuli were generally delivered about 80–90 ms after the end of the time-window from which the stPLV had been estimated (for details see Appendix B, supplementary Figs. S1, S2). This should not cause a relevant problem, since stPLV is evolving more slowly than instantaneous phase — at the very least because it is by construction a moving window average.

### 2.6 Processing of EMG into the response variable

Our aim here was to investigate the influence of real-time single-trial FC on CsE. FC was quantified by real-time stPLV, CsE was quantified by the MEP amplitude in the right FDI muscle. The MEP amplitude was estimated as the range (peak-to-peak) of the [0.020s,0.040s] window of the EMG after each pulse. EMG was also recorded from the right APB (standard procedure in the lab; here used for checking preinnervation).

To remove trials with preinnervation in the EMG, half a second of EMG before each TMS pulse (in the window [−0.505s,−0.005s] w.r.t. the pulse) was linearly detrended, and the range of the detrended signal was computed. If this range exceeded 50μV in APB or FDI (or both), the trial was considered to be affected by preinnervation, and thus removed from the analysis.

MEP amplitudes from FDI were nonlinearly transformed in two steps to get the residuals of the fit models to be (roughly) normally distributed: Firstly, the fourth root was taken (which is bijective on the positive real numbers), and then the moving window median of the result was subtracted (window size: 301 trials, centered on the trial from which to subtract), to combat slow drifts in the MEP-amplitudes, due to e.g. varying drowsiness or head position. These transformed responses were then transformed into z-scores within each session. I.e., the response variable of trial j is computed from the raw MEP-amplitude ${\mathrm{MEP}}_{j}$ as follows:

$$\alpha_{j}=\sqrt[{4}]{{\mathrm{MEP}}_{j}}$$

$$\beta_{j}=\alpha_{j}-\mathrm{median}{\left\{\alpha_{k}\right\}}_{k\in {\mathrm{window}}_{150+1+150}\left(j\right)}$$

$${ResponseFDI}_{j}=\frac{\beta_{j}-\mu }{\sigma }$$

where μ and σ are the mean and standard deviation of the $\beta_{j}$ for the given session.

### 2.7 A priori statistical analysis

Statistical analysis was performed in R (version 4.2.2) using the linear-mixed effects package lme4 (version 1.1.31), the applied regression package car (version 3.1.1), as well as ggplot2 (3.4.1) and sjPlot (2.8.12) for plotting/data and model inspection, and MuMIn (1.47.1) to retrieve the effect size.

To evaluate the main hypothesis, we fitted a linear mixed effects regression (Lmer) model, to account for the remaining inter-subject variability. The Lmer model was formulated as:

(3) ResponseFDI∼1+Condition+(1+Condition|Subject)

in Wilkinson notation (Wilkinson and Rogers, 1973). The categorical fixed effect Condition takes the two values low and high. Since we assumed that different subjects may show different effects of Condition, we also added a random slope.

We further checked the result on the individual level with one-sided Wilcoxon rank sum tests. This splits the participants into ‘responders’ (individual p<0.05) and ‘non-responders’ (individual p>0.05). We checked whether the responders differed from the non-responders in some parameters of their EEG, and/or in their baseline MEPs.

### 2.8 Post-hoc statistical analysis

In the *post hoc* analysis, we tested further explanatory variables that were not included in the main hypothesis, but are commonly related to CsE (Karabanov et al., 2021, Madsen et al., 2019, Hussain et al., 2019): Firstly, we checked whether the spectral power of the μ-band of the C3- and C4-Hjorth signals predicted MEP amplitude. In the most extreme case, it may be that stPLV is merely an indirect way of estimating (a correlate of) the band power, and does not add any information beyond it. Secondly, the time since the last pulse (ISI) was considered as a fixed factor, again to disentangle its effect from the yet hypothetical effect of functional connectivity on CsE. Thirdly, we inspected the cstPLV, stPLV and istPLV computed from the real-time phase-estimate windows that were logged during the experiment. By checking whether the argument of the cstPLVs was (strongly) biased towards 0° (or 180°), we assessed whether there was a strong influence of volume conduction on the stPLV and thus the conditions. Further, the actual stPLV and istPLV values were used as predictors instead of Condition, in *post hoc* Lmers.

To assess the spatial specificity of our results, we also ran the statistical analysis with *post hoc* estimated FC between the C3-Hjorth signal (stimulated, left M1) and each of the following regions: premotor cortex ipsilateral to the stimulated M1, contralateral premotor cortex, ipsilateral occipital cortex, and contralateral occipital cortex.

Lastly, we checked whether there was a relationship between our findings and instantaneous phase of the C3- and/or C4-Hjorth signal.

Spectral power in the μ-band in both left and right M1 was estimated with Matlab’s bandpower function in the 500 ms before each TMS pulse ([−0.505s,−0.005s], shifted to avoid the effects of the TMS-artifact, linearly detrended). The power was log-transformed and z-scored within session within subject, to focus the *post hoc* results on intra-individual variability. The resulting z-score was added as a predictive variable to the Lmer model, which thus becomes:

(4) ResponseFDI∼1+Condition∗MuPowerR∗MuPowerL+(1+Condition|Subject)

In further exploration, the fourth root of the difference of the ISI from the minimum observed ISI was added as a fixed effect with interaction terms (see supplementary material Appendix D), because it has been previously reported that ISI can be predictive of MEP amplitude (Karabanov et al., 2021). We used likelihood ratio (LR) tests to assess whether each included effect improved the model. The constructed models were compared using the Akaike information criterion (AIC) (Akaike, 1974). We pruned insignificant effects from the maximal model, and checked whether the resulting model has a better AIC, and whether such pruning was justified by likelihood ratio tests. The resulting summary model, found by AIC and LR tests is given in Eq. (5).

Further, the stPLV and istPLV were added to the summary model in the place of Condition, to compare the results from the discrete conditions and these continuous predictors. When assessing the spatial specificity of our results, the summary model was likewise modified to include the *post hoc* stPLV between left M1 and the respective other region, and the μ/α-power from that region instead of the power of the C4-Hjorth signal.

#### 2.8.1 Spatial specificity

We checked if our results were spatially specific to the left-M1–right-M1 network, by comparing the results from C3/C4-Hjorth to pairs of C3-Hjorth and occipital and premotor regions in both hemispheres. Signals from these regions were extracted with Hjorth montages over the respective region (see supplementary Table E.2). Since the stPLVs between C3-Hjorth and the locations other than C4-Hjorth have not been computed in real-time, we computed the stPLVs *post hoc*, and compared the results against the results from *post-hoc* stPLVs between C3- and C4-Hjorth. *Post hoc* stPLVs were computed from the same 500 ms window before the TMS pulse as μ/α-power ([−0.505s,−0.005s]), and therefore from a (somewhat) different window than the real-time stPLVs.

#### 2.8.2 Relation to instantaneous phase

To relate our findings to prior literature on the modulation of CsE by μ-phase, we investigated whether single-trial FC is redundant to the instantaneous phase of the μ-rhythm, or complementary to it: First, we checked whether the conditions defined by real-time stPLV also coincide with some pattern of instantaneous phases in the C3- and C4-Hjorth signal. It could, e.g., be hypothesized that in high condition trials, the two signals will generally have the same phase (not merely a constant phase shift). To assess this, we evaluated the very last phase-estimate in the window used for the real-time stPLV (logged during the experiment), for the C3- and C4-Hjorth signals – i.e. the phase closest to the TMS stimulus. Thus, we obtained a pair $\left(\varphi_{\mathrm{C3}}^{\left(t\right)},\varphi_{\mathrm{C4}}^{\left(t\right)}\right)$ for each trial t. The number of trials falling into 2D phase bins was then counted across all participants, yielding 2D histograms for the two conditions, which were then also contrasted.

Secondly, we estimated the instantaneous phase shortly before the TMS pulse *post hoc*: We retrieved a sufficiently long window of the raw C3 and C4-Hjorth signals ([0.605s,0.005s] before the TMS pulse). The signal in this window was linearly detrended, lowpass-filtered (FIR, order: 50, cutoff frequency: 250 Hz), downsampled to 500 Hz, and bandpass-filtered to the μ range (FIR, order: 200, band: 8–13.5 Hz). Whenever applying spectral filters in post-hoc analysis, we used zero-phase digital filtering as implemented by Matlab’s filtfilt function. This avoids shifting the phase of the signal.

The instantaneous phase at the end of the window (5 ms before the TMS pulse) was then determined with the phastimate algorithm (Zrenner et al., 2018, Zrenner et al., 2020).

To use these C3- and C4-Hjorth μ-phase estimates as predictors in a Lmer model, we added the cosine and sine of either phase as fixed effects (circular–linear regression (Cox, 2006, Zrenner et al., 2020)). For illustration, consider a simpler model that predicts a linear response variable by, e.g., C3-Hjorth phase $\varphi_{\mathrm{C3}}$:

$$Response=Intercept+\underset{s\cdot \cos\left(\varphi_{\mathrm{C3}}+\delta \right)}{\underset{︸}{a\cos\left(\varphi_{\mathrm{C3}}\right)+b\sin\left(\varphi_{\mathrm{C3}}\right)}}\mathrm{for}\;\delta =\mathrm{atan2}\left(-b,a\right),\;s=\frac{a}{\cos\left(\delta \right)}$$

The estimated effects a and b thus can be transformed into the amplitude s and the phase-shift δ of a joint sinusoid. Importantly, the cosine/sine term hence cannot be treated in isolation: The effects always have to be considered in pairs, as do their interactions with other fixed effects (e.g., power). We here thus added the C3- and C4-Hjorth phase exploratively, and then pruned the resulting models to see whether we observe some interaction with Condition (or stPLV/istPLV). Since we always have to include a pair of fixed effects (for both main effect and interaction terms), we tested whether adding this pair yields a significant improvement by performing a likelihood ratio (LR) test of the model with both cosine and sine term included against a null-model without those terms.

## 3 Results

### 3.1 Main statistical results

**Fig. 2:**
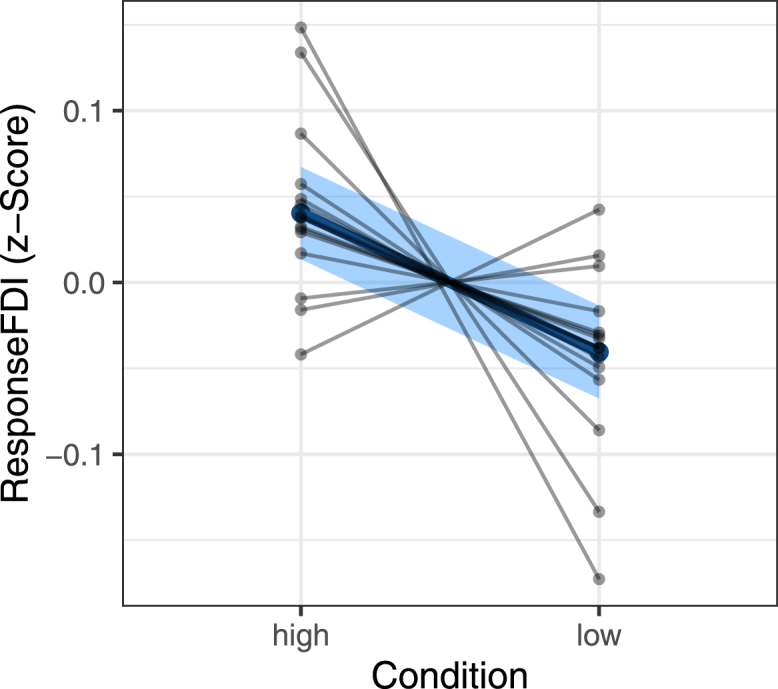
Main result of the primary Lmer model (Eq. (3)): the response (z-score, derived from FDI-MEP-amplitude, see methods) is significantly higher in high condition than in low condition on the group level (blue). This varies on the individual level, as shown by the individual mean responses (black dots): Three participants have a difference of mean response to the conditions that is opposite to the expected. The confidence intervals of the fit are given in light blue, whereas the standard deviations are not given for the individual responses. As ResponseFDI is a z-score, these standard-deviations are all around 1 – contrast this with the very small effect.

The main hypothesis (response is larger in high than in low condition) holds, under the first linear mixed-effect model (Eq. (3)) with p=0.0007 (under Wald $\chi^{2}$-test, W(1)=11.47). Thus, beyond individual variability in the differential response to the condition, there is a significant difference in response between high and low conditions on the group level. As shown in Fig. 2, triggering during the high condition indeed yields larger MEP amplitudes than during low condition. As also displayed in that figure, the effect is not present in all participants, and is overall small: The effect size is $R^{2}\approx 0.0016$ (computed in R via MuMIn::r.squaredGLMM).

Based on pre-innervation, 20% of trials were removed on average (median: 16%), though for one outlier, 71% were removed. Statistics were robust against excluding this participant. For all other subjects, less than a third of trials were removed.

We further checked the presence of the effect of condition on the individual level, by non-paired, one-sided Wilcoxon rank sum tests: 8 of the 15 participants showed a significantly larger MEP amplitude in the high condition. On the session level, which naturally has a smaller sample-size, 2 of 15 showed a significant effect in session 1, and 5 showed a significant effect in session 2 (Table 1). The effect is thus unstable across sessions within the same participant.

**Fig. 3:**
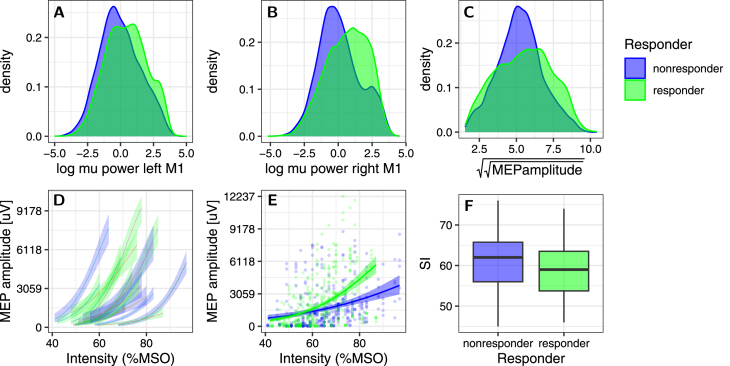
Contrasting ‘responders’ (significant result in Table 1 S1+S2; plotted in green) and ‘non-responders’ (no significant result in Table 1 S1+S2; plotted in blue): A μ-power from the left M1 (logarithm, but not z-scored) in responders vs. non-responders. B μ-power from the right M1. We observe that responders have significantly higher μ-power. C Distribution of the fourth root of the MEP-amplitude during the experiment (across all stimulation trials not excluded due to pre-innervation; not z-scored!). Non-responders have a narrower distribution of Responses (fitting their flatter I/O-curves). D Individual fits of the MEP-I/O-curve (x: stimulation intensity in %MSO, y: raw MEP amplitude (peak to peak)), colored by responder/non-responder. There is a tendency for the non-responders to have flatter I/O-curves somewhat shifted towards higher intensities. E Population level fits of the I/O-curves for responders/non-responders. The non-responders show a flatter I/O-curve on the population level. F Stimulation intensities (SI) used for each participant in each session. Although the non-responders may show flatter I/O-curves, they were stimulated at similar intensities as the responders (no significant difference).

We grouped the participants by the individual Wilcoxon-rank-sum-test results into the 8 responders and 7 non-responders. We have identified two properties that differentiate responders from non-responders, shown in Fig. 3. Firstly, responders have a slightly, but significantly higher μ-power in both C3- and C4-Hjorth ($p<2.2\cdot 10^{-16}$ under Wilcoxon rank sum test for both C3- and C4-Hjorth montages respectively; Fig. 3A/B).

We further checked whether there are differences between responders and non-responders in baseline MEP amplitude. For this, we inspected the MEP-I/O-curves obtained by 25 brain-state independent TMS pulses in each session (Fig. 3D/E). We find that responders tend to have steeper MEP-I/O-curves, i.e., their MEP-amplitude varies more for the same change in stimulation intensity. In spite of this, the stimulation intensities (SI) used in the actual sessions were comparable between responders and non-responders (no significant difference, Fig. 3F). This is possible because the SI was chosen as the individual inflection point of the I/O-curve, independent of the steepness or absolute MEP-amplitude at that point. Furthermore, non-responders seem to have more ‘stereotypical’ MEP-amplitudes, as seen in the narrower distribution of their MEPs (Fig. 3C). Together, this indicates that the MEPs of the non-responders are less sensitive to modulation, resulting in a flatter I/O-curve, and a stereotypical MEP-amplitude.

**Fig. 4:**
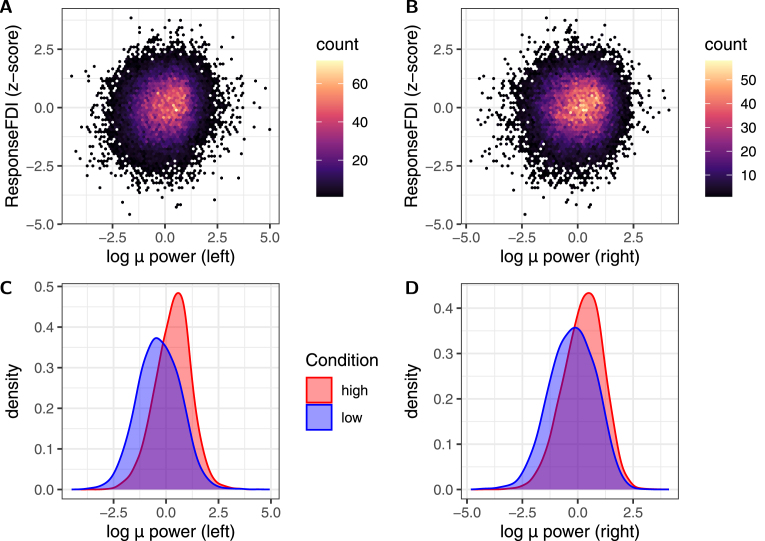
μ-band power is related to the response and condition: In the top row (A, B), the response is plotted against the z-scored logarithm of the bandpower of the (A) left-hemispheric/(B) right-hemispheric signal (in hexbin plots). No strong correlation is visible (Pearson’s R<0.3 for both). In the bottom row (C, D), the distribution of z-scored log power is contrasted in the two conditions (red: high, blue: low). Clearly, high-condition trials tend to have higher μ-power too. This naturally begs the question, whether the difference in MEP amplitude between the high vs. low conditions could not be better explained by the μ-band power — motivating the model given in Eq. (4).

### 3.2 Accounting for μ-band power

Phase-estimates naturally are more reliable when the μ-power is sufficient to produce EEG signals with high signal-to-noise ratio (SNR). Thus, when the bandpower is low, the phase-estimates will be dominated by (mostly) uncorrelated noise from the C3/C4-Hjorth signals. These random phases generally yield low stPLVs in practice. High-power, uncoupled oscillations will also yield low stPLVs — therefore, a low stPLV alone does not suffice to tell whether the observed oscillations are uncoupled or just low in power. When comparing the distribution of the log power between the low and high stPLV conditions, we see that the low condition tends to coincide with low power (Fig. 4). This is true of the power in both hemispheres — with the power moderately correlated between the two hemispheres (Pearson’s r=0.317, $p<2.2\cdot 10^{-16}$). As shown in Fig. 4, both left and right hemispheric power are not visually obviously predictive of the response. Still, both are weakly, but significantly correlated with the response (left: r=0.115, right: r=0.067). Bandpower in the μ-band has been found to be weakly predictive of MEP-amplitude (potentially in interaction with instantaneous μ-phase) (Hussain et al., 2019, Madsen et al., 2019, Karabanov et al., 2021).

This prompted us to check how predictive the high/low condition is for MEP amplitude when also accounting for μ-power (and cross-terms, see Eq. (4)). Adding μ-bandpower to the model shows that indeed, high/low stPLV alone does not predict the response ($W\left(1\right)=0.07,p^{\mathrm{(II)}}\approx 0.7977$ under type II Wald $\chi^{2}$ test), but the interaction-term Condition:MuPowerL with left-hemispheric μ power is predictive of the response ($W\left(1\right)=4.62,p^{\mathrm{(II)}}\approx 0.0316$). Additionally, the Condition shows a significant interaction with right-hemispheric μ power (Condition:MuPowerR, $W\left(1\right)=5.69,p^{\mathrm{(II)}}\approx 0.0171$). The μ-power in either one of the hemispheres also shows a significant main effect (Left: $W\left(1\right)=198.43,p^{\mathrm{(II)}}<2.2\cdot 10^{-16}$, Right: $W\left(1\right)=25.03,p^{\mathrm{(II)}}\approx 5.7\cdot 10^{-7}$), and the powers of the two hemispheres show a significant interaction effect ($W\left(1\right)=13.36,p^{\mathrm{(II)}}\approx 0.0003$). The top-level interaction Condition:MuPowerL:MuPowerR is not significant ($W\left(1\right)=0.02,p^{\mathrm{(II)}}\approx 0.8897$).

**Table 1:**
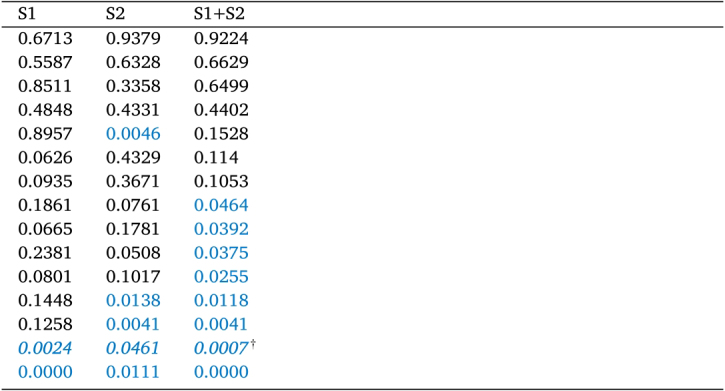
Individual p-values from Wilcoxon rank-sum tests for session 1 (S1), session 2 (S2), and both sessions together (S1+S2). Subjects are ordered by the p-value for both sessions (S1+S2). Note that the sample-size is naturally smaller within the single session compared to taking both sessions together. P-values significant with a 5%-significance-level are highlighted in blue. The outlier subject for whom 71% of trials were removed due to pre-innervation is marked by italicization and dagger.

**Fig. 5:**
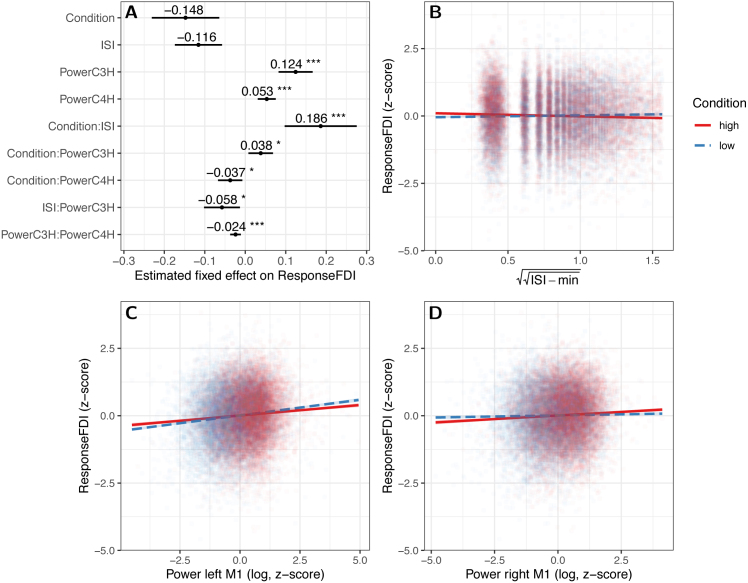
Results of the summary Lmer model (Eq. (5)): A Estimated effects of the fixed factors as a forest plot for the summary model. The estimated value is given above each datapoint, and confidence intervals of the estimates given by horizontal lines. Significance of the effects is indicated by asterisks (‘${}^{*}$’: p<0.05, ‘${}^{**}$’: p∈<0.01, ‘${}^{***}$’: p<0.001). Condition itself lacks a significant main effect, but shows significant interactions with ISI (B), left-hemispheric μ-power (C) and right-hemispheric μ-power (D). B Plot of the interaction of Condition and ISI (somewhat discrete, in steps of 0.1 s due to the implementation): The response in the FDI of the right hand is plotted against the normalized ISI. Each trial is represented by a dot colored by condition (red: high, blue: low). Additionally, the regression-lines (and confidence intervals) are given. C Interaction of Condition with the μ-bandpower of the C3-Hjorth-signal (i.e. the left sensorimotor cortex): For low power, the effect of condition is indeed as expected (lower MEP amplitudes in the low compared to the high condition), but this flips for high power. D Interaction of Condition with the μ-bandpower of the C4-Hjorth-signal (i.e. the left sensorimotor cortex): For high power, the effect of condition is as hypothesized (lower MEP amplitudes in the low compared to the high condition), the opposite holds for low right-hemispheric power. The μ-power in the two hemispheres thus has opposite interactions with Condition.

### 3.3 Summary model

In addition to the μ power, we added ISI as a predictor (see Methods 2.8, and Appendix D). The findings are summarized by the following model, which includes the factors found to have significant effects (by Log-Likelihood-Ratio-tests), and all involved main effects whether significant or not:

(5) ResponseFDI∼1+Condition+ISI+MuPowerR+MuPowerL+Condition:ISI+Condition:MuPowerL+Condition:MuPowerR+ISI:MuPowerL+MuPowerR:MuPowerL+(1+Condition|Subject)

This model is the one with best AIC (${\mathrm{AIC}}^{\text{(5)}}=60891,{\mathrm{AIC}}^{\text{(4)}}=60924,{\mathrm{AIC}}^{\text{(3)}}=61207$).

The estimates for the fixed effects are shown in Fig. 5. Under this model, there is no main effect of Condition ($W\left(1\right)=0.13,p^{\mathrm{(II)}}\approx 0.7204$, estimate: $-0.15\pm 0.04,p^{\mathrm{LR}}\approx 0.0019$ – the LR-test result is inflated, as the interaction terms are not yet present in the corresponding partial models) but, more importantly, Condition does have significant interactions with left-hemispheric somatosensory μ-power ($W\left(1\right)=6.09,p^{\mathrm{(II)}}\approx 0.0136$, estimate: $0.04\pm 0.02,p^{\mathrm{LR}}\approx 0.0077$), right-hemispheric μ-power (W(1)=5.90, $p^{\mathrm{(II)}}\approx 0.0152$, estimate: $-0.04\pm 0.02,p^{\mathrm{LR}}\approx 0.0171$), and inter-stimulus interval ($W\left(1\right)=16.86,p^{\mathrm{(II)}}\approx 0.00004$, estimate: $0.19\pm 0.05,p^{\mathrm{LR}}\approx 2.3\cdot 10^{-7}$).

Inspecting the interaction of left-hemispheric μ-power and condition shows that the effect of high vs low stPLV is flipped to the opposite of the hypothesis for high power: For low μ-power in the C3-Hjorth signal, low stPLV indeed yields a lower MEP-response, but for high power, it is the low stPLV condition that yields higher responses. The response in the low-stPLV condition is more sensitive to changes in μ-power (seen in the slopes in Fig. 5C).

The interaction of condition and right-hemispheric μ-power meanwhile is the opposite: For particularly low right-hemispheric power, low stPLV yields higher Responses, and the high condition is more sensitive to right-hemispheric μ-power (Fig. 5D).

At the same time, long ISIs push high condition trials to lower responses, and similarly, though less strongly, increase the response of low condition trials (Fig. 5B, and Appendix D).

Additionally, there is a significant interaction of ISI and left-hemispheric μ-power ($W\left(1\right)=6.46,p^{\mathrm{(II)}}\approx 0.0110$, estimate: $-0.06\pm 0.02,p^{\mathrm{LR}}\approx 0.0110$), and right and left-hemispheric μ-power show a significant interaction ($W\left(1\right)=12.71,p^{\mathrm{(II)}}\approx 0.0004,p^{\mathrm{LR}}\approx 0.0021$). Lastly, the main effects of the μ-powers also remain significant (left: $W\left(1\right)=190.03,p^{\mathrm{(II)}}<2.2\cdot 10^{-16}$, estimate: $0.12\pm 0.02,p^{\mathrm{LR}}<2.2\cdot 10^{-16}$; right: $W\left(1\right)=21.86,p^{\mathrm{(II)}}\approx 2.9\cdot 10^{-6}$, estimate: $0.05\pm 0.01,p^{\mathrm{LR}}\approx 1.1\cdot 10^{-6}$).

**Fig. 6:**
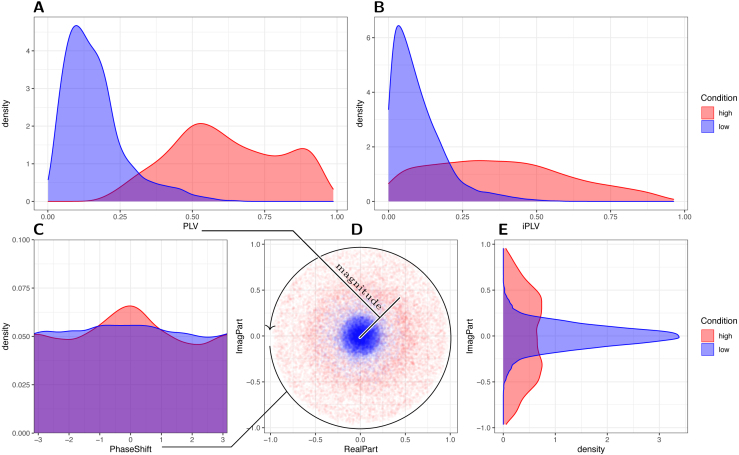
Distribution of measures of functional connectivity between left and right M1, in the low (blue) vs high (red) Condition across all participants: A stPLVs have overlap between the two conditions due to the condition-criteria being dynamic and individual. B imaginary part of stPLV (istPLV) shows more overlap between the conditions. The istPLV follows a fairly flat distribution in the high condition, with many trials having low istPLVs. C Distribution of the argument of the complex stPLV — where a bias towards zero and ±π indicates volume conduction artifact. In the high condition, there is some bias towards 0 which may explain the bigger difference between stPLv and istPLV in the high condition compared to the low condition. Still, the cases with $\pm \frac{\pi }{2}$ phase-shift are not drastically underrepresented. D Scatter plot of the complex stPLVs for each trial in the complex plane. Again, there is no strong bias towards the real axis. E Density of the imaginary part, rotated to highlight the identity of the y-axis with D. Taking the absolute value of the imaginary part yields B.

### 3.4 Influence of volume conduction

**Fig. S3:**
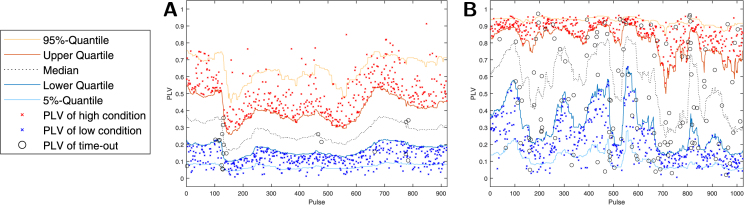
Example time courses of the dynamic condition criteria evolving over the course of the session. The deciding real-time stPLV is plotted for each trial (blue x for low condition, red x for high condition). The orange line indicates the threshold for the high condition (upper quartile of the dynamic empirical stPLV distribution), the blue line indicates the threshold for the low condition (lower quartile). Additionally, the median is plotted as the black dotted line, the 5%-quantile as the light blue line, the 95%-quantile as the yellow line, and the PLV during time-out pulses as black circles. A A participant for whom both criteria are fairly stable, is shown (average case). B A participant with particularly unstable criteria is shown. Observe that there are many more time-out trials on the right, which tend to be particularly common (see steep change in left plot) when the criteria have to change a lot.

We used the simple stPLV to quantify FC in real-time, which can be distorted by volume-conduction effects. To assess the influence of volume conduction on our findings, we inspected the distributions of the stPLVs and related measures: For each trial, the stPLV and imaginary part of stPLV (istPLV), and the underlying complex stPLV (cstPLV) were computed *post hoc* from the logged real-time phase-estimates. As the condition criteria are individual and dynamic, the distributions of stPLV in high and low condition overlap (see Fig. 6, and for examples of the dynamic criteria see supplementary Fig. S3). While the istPLV is generally low in the low condition, it follows a quite flat distribution in the high condition, with stronger overlap between the conditions than for stPLV (Fig. 6).

The reason for the flat distribution of the istPLVs in the high condition is the relative abundance of low and mid magnitude stPLVs in the high condition. To understand this, we first explore two extreme example cases:

**(a)** Let the stPLVs in the high condition all be 1 (or close to one), i.e. all the cstPLVs sit on the unit circle in the complex plane. If the argument of the cstPLVs is uniformly distributed (arg(C)∼Unif([−π,π))), then the imaginary part will have a distribution strongly skewed towards values near ±1, and imaginary parts of ≈0 will be relatively rare. To get an istPLV-distribution as seen in Fig. 6B, the argument would have to be strongly biased towards 0°/180° – indicating a strong distortion of the conditions by volume conduction.

**(b)** Let the cstPLVs be uniformly distributed in the unit disk in the complex plane. The imaginary part will have a distribution with more weight on small absolute values (near zero), and higher values will be rarer, the istPLV will thus be more probable to take small values. Additionally, the stPLVs will follow a linear probability density function (PDF given by f(x)=2x) to indeed produce a uniformly filled circle (Weisstein, 2022). Note that this scenario, although differing from the actual distributions found here, does produce an istPLV-distribution biased towards low values, without the argument of the cstPLvs being biased towards 0°/180°.

We here see that the istPLV-distribution does favor low istPLVs, and as shown in Fig. 6D, the cstPLVs are clearly not distributed on the unit circle, nor uniformly in the unit disk. However, this is not due to a dramatic over-representation of 0°/180° phase-shift among the trials, as is seen in Fig. 6C, although there is a bias towards zero-phase-shift in the high condition. This bias indeed indicates some contamination of the stPLV and thus condition by volume-conduction. The individual participants show varying degrees of such a bias, with no clear outliers. Only one participant shows a bias more towards 180°. Running the analysis after removing the two participants with the strongest bias towards 0° yields roughly the same results as running the analysis on the whole population (though with different p-values, and naturally less statistical power): On the remaining 13 participants, there is no main effect of condition ($W\left(1\right)=0.0001,p^{\left(II\right)}\approx 0.9923$), but it shows a significant interaction with left hemispheric μ-power ($W\left(1\right)=5.75,p^{\mathrm{(II)}}\approx 0.0165$), right hemispheric μ-power ($W\left(1\right)=5.04,p^{\mathrm{(II)}}\approx 0.0248$) and ISI ($W\left(1\right)=16.14,p^{\mathrm{(II)}}\approx 5.9\cdot 10^{-5}$). Moreover, right hemispheric power still shows a significant interaction with left power ($W\left(1\right)=11.18,p^{\mathrm{(II)}}\approx 0.0008$), and the main effect of ISI becomes slightly significant ($W\left(1\right)=3.86,p^{\left(II\right)}\approx 0.0494$). The same picture arises, when the subject that shows a bias towards 180° is additionally removed.

Overall, the results remain stable when removing the subjects with the strongest bias towards 0°/180°. The main effects, which should only be interpreted in the context of the significant interaction terms, shift a bit. The interaction terms (main result) meanwhile remain stable. Volume conduction alone is therefore unlikely to be driving our results.

### 3.5 Continuous stPLV and istPLV

We fit the summary model again, but replacing the categorical Condition with continuous PLV and iPLV respectively. The results are qualitatively similar: The main effects of right and left-hemispheric band power remain significant, and the two still show a significant interaction (W(1)=16.15, $p_{\mathrm{stPLV}}^{\mathrm{(II)}}\approx 5.8\cdot 10^{-5}$, $W\left(1\right)=13.90,p_{\mathrm{istPLV}}^{\mathrm{(II)}}\approx 0.0002$). IstPLV, but not stPLV interacts with left-hemispheric μ-power (W(1)=3.25, $p_{\mathrm{stPLV}}^{\mathrm{(II)}}\approx 0.0715$, and $W\left(1\right)=4.65,p_{\mathrm{istPLV}}^{\mathrm{(II)}}\approx 0.0311$). Contrarily, stPLV, but not istPLV shows a significant interaction with right-hemispheric μ-power (W(1)=4.27, $p_{\mathrm{stPLV}}^{\mathrm{(II)}}\approx 0.0388$, $W\left(1\right)=0.83,p_{\mathrm{istPLV}}^{\mathrm{(II)}}\approx 0.3637$).

Both stPLV and istPLV show a significant interaction with ISI ($W\left(1\right)=13.80,p_{\mathrm{stPLV}}^{\mathrm{(II)}}\approx 0.0002,W\left(1\right)=8.95,p_{\mathrm{istPLV}}^{\mathrm{(II)}}\approx 0.0028$). StPLV does not achieve a significant main effect ($W\left(1\right)=0.99,p_{\mathrm{stPLV}}^{\mathrm{(II)}}\approx 0.3204$), nor does istPLV ($W\left(1\right)=2.44,p_{\mathrm{istPLV}}^{\mathrm{(II)}}\approx 0.1184$). For both, ISI has a significant main effect ($W\left(1\right)=4.86,p_{\mathrm{stPLV}}^{\mathrm{(II)}}\approx 0.0275,W\left(1\right)=4.81,p_{\mathrm{istPLV}}^{\mathrm{(II)}}\approx 0.0283$)

### 3.6 Spatial specificity

We note that the model using *post hoc* stPLVs between C3- and C4-Hjorth is a bit worse than the model using the real-time condition ($p^{\mathrm{LR}}<2.2\cdot 10^{-16},{\mathrm{AIC}}^{\mathrm{posthoc}}=60908>60891={\mathrm{AIC}}^{\mathrm{realtime}}$). In switching to other montages in the place of C4-Hjorth, we firstly find no result for contralateral occipital cortex ($p^{\mathrm{(II)}}>0.1$ for all model terms including μ/α-power from O2-Hjorth and/or the stPLV between C3-Hjorth and O2-Hjorth). For ipsilateral occipital cortex, we only find a weak main effect of the stPLV between C3-Hjorth and O1-Hjorth ($W\left(1\right)=4.60,p^{\mathrm{(II)}}\approx 0.0319$, estimate: −0.05±0.07), but no effects relating to O1-Hjorth α-power.

For premotor cortex, we find a significant interaction effect of the μ-power of C3-Hjorth, and the μ-power of the respective premotor signal across both hemispheres (F3-Hjorth: $W\left(1\right)=5.40,p^{\mathrm{(II)}}\approx 0.0202$, FC3-Hjorth: $W\left(1\right)=13.55,p^{\mathrm{(II)}}\approx 0.0002$, F4-Hjorth: $W\left(1\right)=17.99,p^{\mathrm{(II)}}\approx 2.2\cdot 10^{-5}$, FC4-Hjorth: $W\left(1\right)=17.74,p^{\mathrm{(II)}}\approx 2.5\cdot 10^{-5}$). No other significant effects of terms relating to the premotor regions are found in any of these models (only the remaining C3-Hjorth results).

**Fig. 7:**
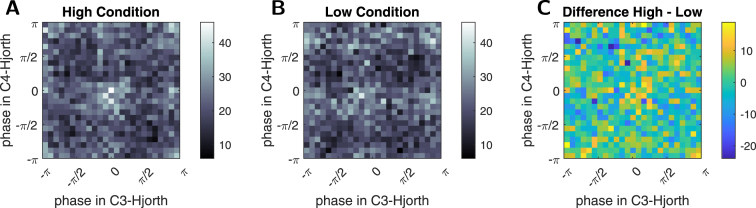
2D Histograms investigating the relation of functional-connectivity condition and instantaneous phase: A For each trial in the high condition, the instantaneous phase at the very end of the window of real-time stPLV estimates of the C3- and C4-Hjorth signal respectively was retrieved (logged during experiment, n=13500), and the combinations of (binned) phases were then counted. B The same was done for the low condition trials (n=13500). C The difference of counts in each bin is given. There is no clear pattern emerging, e.g. there is no clear diagonal increase of depletion of certain phase-pairs in high vs. low condition. There is a slight diagonal pattern, but it is weak. Statistical analysis does not indicate an interaction or redundancy of phase and condition either (see main text). The colormaps differ between A and B versus C, to highlight the different ranges of values in the 2D-histograms.

We therefore find our results to be spatially specific, with only electrodes ipsilateral to the stimulated hemisphere, or located over the bihemispheric motor network, showing echoes of the results for C3/C4-Hjorth.

### 3.7 Relation to instantaneous phase

In relating our findings to prior research into the modulation of CsE by μ-phase, we checked whether the single-trial FC-defined conditions coincided with some clear pattern of instantaneous phases. In a first analysis, using the real-time phase-estimates logged during the experiment (see Section 2.8), we obtained the 2D histograms shown in Fig. 7. Taking the per-bin difference does not indicate any clear difference between the conditions with respect to the instantaneous phase of the bihemispheric μ-rhythm shortly before the TMS-pulse. This first analysis therefore indicates that real-time FC and instantaneous phase are not redundant.

**Fig. 8:**
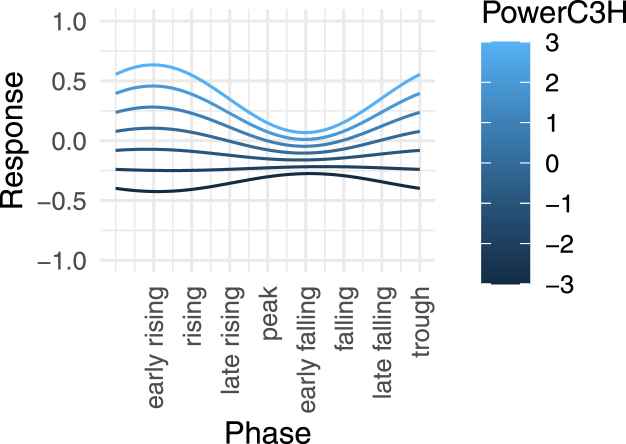
Visualization of the estimated interaction effect of the μ-phase and (log) μ-band power of the C3-Hjorth signal in predicting MEP-amplitude (response): For high μ-band power, the early rising phase is the state of highest excitability, whereas for low μ-band power, the early falling phase is the high-excitability state. These results align with previous findings (Hussain et al., 2019, Zrenner et al., 2023).

To investigate this further, we added the C3- and C4-Hjorth-phase to the Lmer-analysis as fixed effects: We thereby firstly found that right-hemispheric μ-phase (C4-Hjorth) was not predictive, nor did it show significant interactions with any other term. Meanwhile, the μ-phase in the stimulated hemisphere (C3-Hjorth) had a significant main-effect ($\chi^{2}\left(2\right)=116.59,p^{\mathrm{(LR)}}<2.2\cdot 10^{-16}$), and more importantly, showed a significant interaction with μ-power at the same site ($\chi^{2}\left(2\right)=37.73,p^{\mathrm{(LR)}}\approx 6.4\cdot 10^{-9}$). This interaction is shown in Fig. 8: For high μ-band power, the early falling phase is the phase of highest CsE. For low μ-band power, the early falling phase instead is the phase of highest CsE, while the early falling phase under low μ-power is the phase of lowest CsE. Our findings thus agree with prior literature (Hussain et al., 2019, Zrenner et al., 2023).

Instantaneous phase did not interact with Condition ($\chi^{2}\left(2\right)=7.61,p^{\mathrm{(LR)}}\approx 0.1790$), nor with stPLV ($\chi^{2}\left(2\right)=8.66,p^{\mathrm{(LR)}}\approx 0.1236$), nor istPLV ($\chi^{2}\left(2\right)=7.55,p^{\mathrm{(LR)}}\approx 0.1826$). The summary model with the cosine and sine of C3-Hjorth μ-phase main-effects and interactions with C3-Hjorth μ-power added as fixed factors achieved the best AIC of any model tested (AIC=60744), without suffering from the high collinearity of predictors that the previous maximal model suffered from.

## 4 Discussion

We here demonstrated that single-trial functional connectivity between TMS-targeted left and the contralateral right M1 can define a state of high corticospinal excitability that can be targeted with real-time EEG-TMS. The main hypothesis thus could be verified. Closer inspection showed that both left-hemispheric μ-power and inter-stimulus interval (ISI) interacted significantly with the high and low functional connectivity conditions. Furthermore, the conditions defined by the single-trial phase-locking value (stPLV) are non-redundant with instantaneous phase and, therefore, single-trial functional connectivity is here demonstrated to complement the hitherto established real-time markers of high corticospinal excitability (μ-power (Madsen et al., 2019, Hussain et al., 2019, Thies et al., 2018), ISI (Karabanov et al., 2021), instantaneous μ-phase (Zrenner et al., 2018, Hussain et al., 2019)).

### 4.1 Interpretation

Interpreting functional connectivity estimated from EEG is generally difficult (Bastos and Schoffelen, 2016). We here used the single-trial version of an undirected connectivity measure that could be affected by volume conduction. As we showed in Fig. 6 and by using istPLV as a predictor *post hoc*, while volume conduction does affect the high condition, it did not dramatically skew our results. The conditions thus capture times of high/low single-trial functional connectivity between the C3- and C4-Hjorth signals, but do not inform us about the direction in which signaling/communication may occur. For instance, high functional connectivity *could* indicate strong inhibitory interactions between the two. Without knowing the direction of the interaction, this might well mean that the stimulated left M1 is inhibited (and poorly excitable) in the high condition.

Indeed we here found that the functional connectivity between the left and right M1 alone gives a very incomplete view of how corticospinal excitability is regulated: left hemispheric power, right-hemispheric power, and inter-stimulus interval all flip the effect of the FC conditions (Fig. 5). Similar flipping of high excitability states due to μ-power has been observed for μ-phase (Hussain et al., 2019), highlighting the importance of either including μ-power as a predictor or controlling it to be in a desired range (e.g., above a minimum power criterion (Stefanou et al., 2019)).

High μ-power in the left M1 firstly indicates that the local neuronal population is firing synchronously — perhaps indicating an opportunity for effective TMS of the whole population at once. High μ-power in the C3-Hjorth signal coincides with high MEP amplitudes (Fig. 5, and Thies et al., 2018, Karabanov et al., 2021). Secondly, high μ-power naturally occurs when no motor action is performed or imagined. Interpreting the interaction of functional connectivity and μ-band power (Fig. 5C), the combination of high left-hemispheric μ-power and high functional connectivity between left and right M1 might thus mean that the right M1 is currently inhibiting the left M1, yielding a lower TMS-response than in the absence of communication between the two hemispheres (low FC). Notably, the power of the unstimulated hemisphere shows the opposite interaction with Condition: For high μ-power in right M1, high FC coincides with higher responses. This could indicate that, if the two M1s are communicating (high FC), whichever M1 shows stronger (inhibitory) μ-power may be receiving inhibitory input from the other M1.

It is however possible that the interaction of left hemispheric μ-power with the condition has more of a technical than a physiological reason: The TMS-coil often produces considerable 50 Hz noise in the closest electrode (one of the C3-Hjorth montage electrodes), which – sometimes dramatically – decreases the reliability of the real-time phase-estimates. The phase-estimates then become visibly noisy, deviating from the expected saw-tooth-waveform. A certain μ-power is thus needed to get a sufficient SNR for reliable phase-estimates (Zrenner et al., 2020). Since low μ-power will generally mean that the phase-estimates are (more) dominated by often uncorrelated noise and thus, that the stPLV is low, low condition trials tend to have lower μ-power, and are more sensitive to the μ-power.

**Fig. S4:**
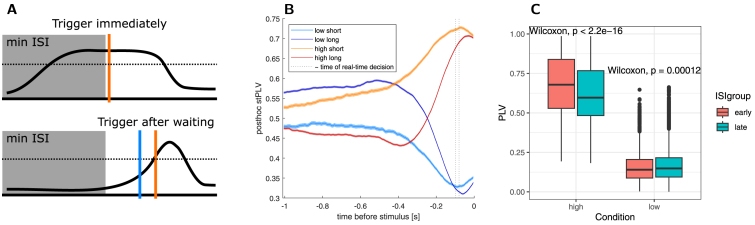
Inspecting the role of ISI on our dataset: A There are two groups of trials, those with ISI<2.35 s (top), and the rest (bottom). In the short-ISI trials, the first stPLV (timing marked in orange) that was computed after the enforced minimum ISI (grayed out area) already matched the awaited next condition (here: high condition). In these trials, it is possible that there was a sustained high FC for longer than 100 ms before the pulse. In the long-ISI trials (bottom), there was at least one stPLV (blue mark) before the deciding stPLV (orange mark), which did not match the awaited condition. Here, the stPLV at the blue mark was below the high-condition threshold. This means that in the long-ISI trials, there cannot be a period of sustained high FC longer than 100 ms before the deciding stPLV. B Pre-stimulus FC timecourses (population average) for the high and low conditions, and short (ISI<2.35 s) vs. long (ISI>2.35 s) ISI trials. The stPLVs were estimated *post hoc* from 500 ms windows. The distance of the right edge of that window and the stimulus is shown on the x-axis. The approximate range of the time of the decisive stPLV is indicated by the dotted gray lines. C The logged decisive real-time stPLVs were compared between short (early) and long (late) ISI-groups within condition. For both conditions, there is a significant difference between the short-ISI and long-ISI trials in real-time stPLV (tested with two-sided Wilcoxon rank sum tests).

The reason for the interaction of ISI and Condition is unclear: This finding is robust against excluding the trials with very short ISIs and against excluding the very long ISIs, and against excluding both at once, but we could not reproduce it on a supplementary brain-state independent dataset that is otherwise comparable (see Appendix D). modified As explained in Appendix D and supplementary Fig. S4, there is a technical difference between the trials with very short ISI and the remaining trials: short-ISI trials can be and frequently are preceded by sustained high/low FC, whereas the non-short ISI trials have to be preceded by a change in FC (crossing of condition threshold). This is due to the waiting procedure (Fig. 1) in combination with the enforced minimum ISI of 2 s. Indirectly, this also led to a slight difference in the deciding stPLV between short and non-short ISI trials (supplementary Fig. S4). Although it does not seem to be responsible for the unexplained ISI-results, future studies will thus benefit from avoiding this pitfall. This could be done, e.g., by waiting for the first stPLV that matches the awaited condition and is preceded by at least one stPLV not matching the awaited condition — thus, the first computed stPLV after the minimum ISI would be excluded from triggering TMS-pulses. Before interpreting this more, the interaction of functional connectivity and ISI should be replicated in subsequent research, and for now should be taken as an unverified result of the *post hoc* analysis.

### 4.2 Limitations

One key problem of our setup is that it introduces a fairly long delay between the data-window used to estimate an stPLV, and the stimulus — at about 80–90ms. This makes it possible for the network’s connectivity to have already changed to another state when the stimulus is delivered. From the *post hoc* analysis by which we estimated the delay (supplementary material), we can see that indeed the separation of the conditions is partially degraded at the time of the stimulus — but that the conditions still clearly differ in functional connectivity (supplementary Fig. S2). The main reason for this problem is that we performed parts of the processing on the (weak) Windows-PC in the laboratory, which had to retrieve the phase estimates from the real-time EEG-processing device. Future experiments will benefit from moving this processing to the real-time-device, removing the slower lab-PC from the loop.

MEPs give only a very noisy estimate of corticospinal excitability, as they are highly variable due to a large range of cortical, and especially peripheral influences (Cuypers et al., 2014). They are therefore not good single-trial estimators (Cuypers et al., 2014), which is why we need a large number of trials to investigate the fairly subtle influence of single-trial FC on CsE. Naturally then, the remaining unexplained variance is large, the conditions overlap greatly in the response, and the effect size is small.

We observed a difference in mu-power between ‘responders’ and ‘nonresponders’. This difference may indicate that the inclusion criterion was too lenient. In particular, the difference in C4-Hjorth μ-power, which was not considered separately for the inclusion criterion, is larger. In principle, this is not a very surprising finding, as higher μ-power means a better SNR for phase estimation and subsequently FC-estimation. If the phase-estimates are too noisy (low SNR by low μ-power), the two conditions cannot be meaningfully distinguished. This is a weakness that our study shares with phase-targeted real-time stimulation, and may be a hindrance in applying FC (and phase) based brain-state dependent stimulation to stroke patients, who may have worse SNR.

Similarly, although we already pre-selected the participants, only including those with a sufficient μ-SNR, we still had immediate problems with the SNR, as the coil can introduce substantial 50 Hz noise in the C3-Hjorth signal. This 50 Hz noise deteriorated real-time left-hemispheric phase-estimates – sometimes dominating over physiological signals. We unfortunately could not solve this issue in the experiment with the particular coil and stimulator used.

Furthermore, EEG is susceptible to noise from superficial physiological sources, like muscles: One participant showed strong muscle artifact in the Hjorth-montages in the first session, yielding a highly insignificant result. Giving the participant more detailed feedback on this muscle artifact alleviated it almost entirely, yielding a highly significant result in session two — but still an insignificant result overall. Similar approaches did not work in all participants however, although all the participants were healthy and co-operative.

We here did not control μ-power to be above a minimum power threshold to enforce some minimal SNR. While this does allow us to study the interaction of power with FC over the whole range of power expressed in the subjects, this negatively affects the reliability of the phase-estimates (Zrenner et al., 2020) – and thereby also the reliability of our findings for low power.

The concern that motivates using iPLV over PLV is that PLV can easily be dominated by volume conduction (Bastos and Schoffelen, 2016). While stPLV can be inflated by volume conduction, Hjorth montages over two spatially well-separated electrodes should be able to (mostly) remove this effect, as the signal from a distant source will be sufficiently blurred to arrive roughly the same in all five electrodes. Fittingly, we find some, but no drastic contamination by volume conduction. Even in the subjects with the strongest bias towards 0° phase-differences, all phase-differences are sampled and represented by a reasonable number of trials.

When the nodes of the targeted networks are closer to each other, the influence of volume conduction is likely to only increase, so using istPLV or other volume-conduction-robust measures is advisable (Marzetti et al., 2019).

**Table A.1:** Percent of retained trials after removing pre-innervated trials, for each subject. Subjects are ordered in ascending order with respect to the percentage of retained trials. Subject 12 is an outlier with only 29.5% of trials showing no preinnervation.

Subject	12	06	04	01	03	08	13	09	07	05	15	10	02	14	11
% trials left	29.5	69.2	74.9	77.9	82.0	82.5	83.1	84.1	85.8	85.8	86.9	89.2	89.4	90.9	91.1

### 4.3 Relevance

The treatment of various brain network diseases (such as motor stroke (Guggisberg et al., 2019)) is expected to benefit from treatments using a network perspective (Uhlhaas and Singer, 2012, Ziemann et al., 2019). We here presented first experimental evidence that a very simple network-measure (dynamic connection strength, as measured by single-trial FC) can be targeted with real-time EEG-TMS to get differential TMS-responses. The motor system is damaged in motor stroke, with changes in the structural and effective connectivity between the nodes (Grefkes and Fink, 2011, Rehme et al., 2011). Depending on the damage caused by the stroke, the path to recovery may involve overcoming a maladaptive inhibition of the ipsilesional motor cortex by the contralesional M1 (interhemispheric competition) (Nowak et al., 2009), or vicariation — where the contralesional hemisphere supports or takes over tasks of the ipsilesional hemisphere (Di Pino et al., 2014, Guggisberg et al., 2019). It is plausible that the influence of real-time functional (or effective) connectivity on corticospinal excitability could be used as a diagnostic biomarker to classify patients to select appropriate treatment — which is important, as the same treatment aimed at reducing the excitability of the contralesional M1 may be beneficial to patients with maladaptive interhemispheric inhibition, but detrimental for patients with vicariation (Guggisberg et al., 2019).

Beyond diagnostics and treatment-group selection, (EEG-)TMS promises to serve as a therapeutic tool inducing plasticity in the stimulated network (Ziemann et al., 2019, Guggisberg et al., 2019, Di Pino et al., 2014, Baur et al., 2020). States of particularly high (or low) excitability (and/or of disinhibition) have been shown in both local field potential studies (Huerta and Lisman, 1995), TMS (Cash et al., 2016) and EEG-TMS studies (Zrenner et al., 2018, Baur et al., 2020) to be promising candidates for inducing plastic changes in a defined direction. Targeting times of particular functional (or effective) connectivity states in the motor network is thus a promising avenue to induce plastic changes within the motor-cortical system.

Adding functional connectivity as another real-time accessible marker of high corticospinal excitability complements previously established markers, in particular phase and power of the μ-rhythm, and may help define a more robust composite biomarker of high excitability states in motor cortex — and potentially opportune windows of network plasticity induction.

## Funding

This study is part of the Connect-to-brain project that has received funding from the 10.13039/501100000781European Research Council (ERC) under the 10.13039/501100007601European Union’s Horizon 2020 research and innovation programme (Grant agreement No. 810377). The work by T. Mutanen has been supported by the 10.13039/501100002341Academy of Finland (Grant No. 321631) and the 10.13039/501100005637Finnish Foundation for Technology Promotion .

## CRediT authorship contribution statement

**David Emanuel Vetter:** Designed the study, Developed the codes, Recruited the patients, Data curation, Conducted the data analysis, Writing – original draft. **Christoph Zrenner:** Designed the study, Developed the codes. **Paolo Belardinelli:** Conducted the data analysis, Provided assistance with interpretation. **Tuomas Petteri Mutanen:** Conducted the data analysis, Provided assistance with interpretation. **Gábor Kozák:** Developed the codes, Provided assistance with interpretation. **Laura Marzetti:** Conducted the data analysis, Provided assistance with interpretation. **Ulf Ziemann:** Designed the study, Provided assistance with interpretation.

## Declaration of competing interest

C. Zrenner declares an equity holding in sync2brain GmbH, G. Kozák declares salary support from sync2brain GmbH as a part-time employee, sync2brain GmbH is a University of Tübingen spin-off that commercializes a variant of the real-time EEG analysis device used in this research. T. Mutanen has received funding for a collaborative research project with Bittium Biosignals Oy (Kuopio, Finland). All other authors declare no competing interest.

## Figures and Tables

**Fig. S5:**
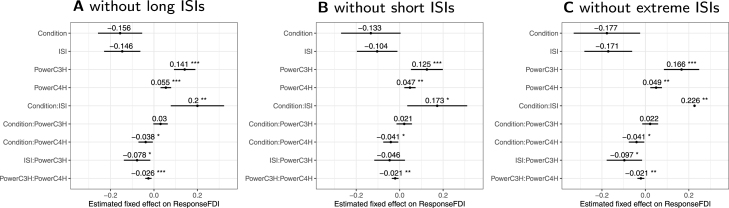
Analogous to Fig. 5A, but with trials excluded based on ISI: A Only on those trials with ISI<4s are included. B Only on those trials with ISI>2.35s are included. C Only on those trials with ISI∈[2.35s,6s] are included (wider range to retain a comparable amount of trials, and not loose too much statistical power). The exact estimates differ, yet the interaction of Condition and ISI remains similar.

**Table E.2:** Regions and corresponding spatial filters used to check the spatial specificity of our results: Two Hjorth montages were used for checking the premotor cortex: One, more posterior and broader, centered on the FC3/FC4 electrode, and one, more anterior and narrower, centered on the F3/F4 electrode. Note that O1/O2 is at the very edge of the cap, thus there is no electrode caudal of O1/O2. We have therefore opted for a partial Hjorth montage around these electrodes. The C3/C4-Hjorth spatial filters are given for completeness.

Area	Ipsilateral (left)	Contralateral (right)
Primary motor cortex	$\mathrm{C3}-\frac{1}{4}\left(\mathrm{FC1}+\mathrm{FC5}+\mathrm{CP1}+\mathrm{CP5}\right)$	$\mathrm{C4}-\frac{1}{4}\left(\mathrm{FC2}+\mathrm{FC6}+\mathrm{CP2}+\mathrm{CP6}\right)$
Premotor cortex	$\mathrm{FC3}-\frac{1}{4}\left(\mathrm{F1}+\mathrm{F5}+\mathrm{C1}+\mathrm{C5}\right)$	$\mathrm{FC4}-\frac{1}{4}\left(\mathrm{F2}+\mathrm{F6}+\mathrm{C2}+\mathrm{C6}\right)$
	$\mathrm{F3}-\frac{1}{4}\left(\mathrm{AF3}+\mathrm{F1}+\mathrm{F5}+\mathrm{FC3}\right)$	$\mathrm{F4}-\frac{1}{4}\left(\mathrm{AF4}+\mathrm{F2}+\mathrm{F6}+\mathrm{FC4}\right)$
Occipital cortex	$\mathrm{O1}-\frac{1}{3}\left(\mathrm{PO3}+\mathrm{PO7}+\mathrm{Oz}\right)$	$\mathrm{O2}-\frac{1}{3}\left(\mathrm{PO4}+\mathrm{PO8}+\mathrm{Oz}\right)$

## Data Availability

Data available for medical/physiological research upon request to the corresponding author. Compliance with EU data protection law (Regulation (EU) 2016/679) is required. An “End User Licence Agreement” (EULA) will be required before data will be released. Data will be shared on a project-specific basis. The code we used to run the experiment, and the analysis pipeline are available on GitHub (https://github.com/connect2brain/MoCsEFC).

## Appendix A Preinnervation

See Table A.1.

## Appendix B Estimating the delay between PLV-window and TMS-pulse

### B.1 Post-hoc delay estimation

We evaluated the delay between deciding stPLV and TMS pulse (Fig. S1) *post hoc*. To this end, we took the recorded EEG in a 500 ms window before the pulse-marker, and computed the stPLV from it: in the raw C3-Hjorth/C4-Hjorth signals, the TMS-artifacts were interpolated linearly, the 50 Hz noise was filtered out with a zero-phaseshift FIR bandstop filter of order 5000, the resulting signal was bandpass filtered between 8–13.5 Hz and then Hilbert-transformed. Then, we computed the difference of the *post-hoc* stPLV from the dynamic criteria (upper and lower quartile) in the respective trial, as logged during the experiment. We then compared the distributions of the difference of the *post-hoc* stPLV from the upper and lower criterion in the low vs. high condition (by one-sided Mann–Whitney-U-tests).

When varying the time between the right edge of the window from which the stPLV was computed *post hoc*, we observe that the separation of the conditions is maximized (p-value from Mann–Whitney-U test is minimized) for a delay of about 80–90 ms (Fig. S2). There are two points supporting this (beyond the approach in itself):

• This offset is consistent across participants, varying around ≈80–100 ms
• Initially, there was a mistake in the experiment-script: Instead of the last 500 ms of the phases (cf. Fig. S1), the first 500 ms were used to estimate the stPLV in real-time. This introduced a 500 ms **forced** delay in addition to any processing delay. When checking this with the above *post-hoc* approach, it gave a delay of ≈600ms – consistent with 500 ms of forced, artificial delay plus ≈90ms of actual delay (see Fig. S2)

For some participants, as seen by the slight dip in Fig. S2 towards zero offset, there seems to be a somewhat consistent pattern of stPLV-switching, making the stPLV from 500 ms before the stimulus somewhat predictive of the stPLV right before stimulation.

## Appendix C Variability of dynamic criteria

See Fig. S3.

## Appendix D The role of the inter-stimulus interval

### D.1 Accounting for inter-stimulus interval

To account for the possible effect of ISI, we fit a Lmer-model with ISI as a fixed factor (and all interactions). Under this model, there is no significant main effect of ISI ($W\left(1\right)=2.37,p^{\mathrm{(II)}}\approx 0.1238$), but there is a significant interaction of Condition and ISI ($W\left(1\right)=15.48,p^{\mathrm{(II)}}\approx 8.33\cdot 10^{-5}$): For long ISIs, high condition trials yield a smaller response than low condition trials. For short ISIs, there is only a tiny difference (in the opposite direction), as shown in Fig. 5. Additionally, there is a significant interaction of ISI and left hemispheric μ-power ($W\left(1\right)=4.86,p^{\mathrm{(II)}}\approx 0.0274$). It is worth noting that this maximal model suffers from collinearity of the predictors, motivating the removal of some of them in the summary model.

#### D.1.1 Role of interstimulus interval

Due to the experimental setup, there is a group of trials with the shortest ISI (ISI<2.35 s), which differs from the remaining longer-ISI-trials: In the experiment, after each pulse, a 2 s minimum ISI was enforced, before the wait for the next condition was started. In all the trials with ISI<2.35 s, the first real-time stPLV that was computed after this enforced minimum ISI, already matched the next condition, and thus, a pulse was delivered immediately. In all other trials (ISI>2.35 s), the real-time stPLV did not match the next condition at least once after the minimum ISI. As illustrated in supplementary Fig. S4, this means that only short-ISI trials can feature a period of sustained high (or low) FC preceding the deciding stPLV and the pulse. We checked whether this is the case by computing the stPLVs *post hoc* in a moving 500 ms window preceding the pulses (Fig. S4).

Indeed, we observe that long-ISI trials tend to be preceded by a period of FC in the opposite direction of the awaited condition. I.e., a long-ISI high-condition pulse was on average preceded by a period of sustained lower-than-average FC, and the FC on average only started to rise shortly before the pulse. Meanwhile, short-ISI pulses were on average preceded by a longer period of FC matching the awaited condition. Additionally, short-ISI trials showed more extreme decisive real-time stPLVs than long-ISI trials (Fig. S4).

Since there is this technical difference between the short and long ISI trials, we tried whether removing the trials with short ISIs (<2.35 s) would change our results. Similarly, we also checked if the results would change when removing trials with particularly long ISIs, and when removing both the short ISIs and particularly long ISIs. An additional reason for these checks is that the high condition shows slightly more very long and very short ISIs than the low condition, which tends to have intermediate ISIs.

The result is summarized in Fig. S5: whether we removed the trials with particularly short ISI, or the trials with particularly long ISI, or removed both, the results did not change markedly. However, with this many trials removed, the statistical power is substantially weakened.

In particular, the interaction term of Condition and ISI remains very stable (Fig. S5). It is therefore not an artifact of the early ISI-trials, nor of very long ISI trials. We thus conclude that the interaction effect between ISI and Condition is not (exclusively) due to the trials with very short ISI (nor due to those with very long ISI, or their combination), but is present among the trials with mid-range ISIs.

### D.2 Supplementary dataset

In trying to elucidate the role of ISI, we consulted an additional brain-state independent TMS dataset (n=18 subjects, one of which also had participated in the main study). In this dataset, TMS had been delivered to the FDI/APB-informed motor hotspot in the left M1, independent of the ongoing brain-state, with ISIs ranging from 2.5 s to 3.5 s (mean: 3 s). Matching the main dataset, the participants were healthy, right-handed volunteers of the same age-group as in the main dataset (university students). Unlike the main dataset, the supplementary dataset contains on average 989 trials (min: 254, second lowest: 728, max: 1196) after rejecting trials affected by pre-innervation. This is a little more than one session of the main experiment yielded, but less than the two sessions of the main experiment.

If the interaction of ISI and stPLV that we observe on the main dataset could be found on the supplementary dataset, we could still not explain it physiologically, but would have evidence that it is not just some technological or statistical fluke.

To that end, we computed the *post-hoc* μ-band stPLV in the [−0.505s,−0.005s] window before each pulse, as well as the μ-power, exactly as done for the *post-hoc* analysis on the main dataset, and ran the same statistical analysis. To make the estimated effects more comparable (for interpretation), we subtracted the minimum ISI found in the main dataset from the ISIs of the supplementary dataset, and not the minimum ISI found in the supplementary dataset.

#### D.2.1 Results on the supplementary dataset

We compare the results of the supplementary dataset with the results of the main dataset using *post-hoc* stPLVs. We found no interaction term of ISI and stPLV on the supplementary dataset, though the estimated effect is of a similar size, only much more uncertain: −0.21±0.44 ($p^{\mathrm{LR}}\approx 0.5125,p^{\mathrm{(II)}}\approx 0.6274$) for the supplementary dataset vs. −0.24±0.08 ($p^{\mathrm{LR}}\approx 0.0040,p^{\mathrm{(II)}}\approx 0.0040$) for the main dataset using *post-hoc* stPLV. This result does not change notably when excluding the four subjects in the supplementary dataset, who would have been excluded from the main experiment due to insufficient μ SNR in the resting state EEG.

We did however find an interaction term of ISI and the μ-power of the C3-Hjorth signal, which also was found on the main dataset, though with a very different estimated effect: 0.62±0.29 ($p^{\mathrm{LR}}\approx 3.6\cdot 10^{-10},p^{\mathrm{(II)}}\approx 0.0272$) for the supplementary dataset vs. −0.07±0.02 ($p^{\mathrm{LR}}\approx 0.0013,p^{\mathrm{(II)}}\approx 0.0013$) on the main dataset. Notably, the models on the supplementary dataset suffer from higher variance inflation due to collinearities between the predictors than the same models on the main dataset.

We thus could not reproduce the ISI-related results of the main dataset on the supplementary dataset.

## Appendix E Spatial specificity

See Table E.2.
